# Action at a Distance: Theoretical Mechanisms of Cross-Dendritic Heterosynaptic Modification

**DOI:** 10.1523/ENEURO.0419-23.2023

**Published:** 2023-11-10

**Authors:** Sahil Moza

**Affiliations:** Department of Organismic and Evolutionary Biology, Harvard University, Cambridge, MA 02138

## Abstract

**Highlighted Research Paper:** T. Moldwin, M. Kalmenson, and I. Segev, “Asymmetric voltage attenuation in dendrites can enable hierarchical heterosynaptic plasticity.” *eNeuro* (2023).

Neurons as integrate-and-fire points are a convenient abstraction for machine-learning and network models of circuit-dynamics in the brain. However, the diversity of elaborate neuronal morphologies, spatial distributions of ion channels, compartmentalized chemical computations and plasticity mechanisms paint a picture of individual neurons as an astounding, intricate web with several control-knobs and set points. Such properties endow neurons with several computational opportunities. In a recent article, Moldwin et al. (2023) consider the computations that lay in the diversity of the distributions of synapses across dendrites. What effect does the organization of synaptic inputs on the dendrites of pyramidal neurons have on synaptic plasticity? Moldwin and colleagues analyze this question theoretically and generate several testable predictions in the context of heterosynaptic plasticity.

The authors explore the interactions between a calcium-based synaptic-plasticity rule with passive dendritic voltage decay first using a ball-and-stick model, and then, a hierarchical dendritic-tree model based on a cortical layer-5 pyramidal neuron. They find that the spatial position of synaptic inputs on the dendritic tree can strongly affect both the direction and the degree of heterosynaptic plasticity. Cable theory predicts that soma and its attached dendrites act as a “current sink.” The overall leak conductance increases with membrane surface area from distal dendrites to soma. This causes a reduction in transfer resistance, and consequently, a steeper attenuation of voltage from distal to proximal sites than in reverse. Thus, the signal from depolarizing synaptic inputs at distal dendrites attenuates steeply toward the soma because of this crucial factor called asymmetric voltage attenuation (AVA). The authors consider a reduced model of neurons as a branching tree with three branching points. They subsume the complex dendritic arbor into the soma and increase its diameter to match the distance-dependent decrease in transfer resistance. Another crucial factor is the higher dendritic input resistance, which amplifies the depolarization caused by synaptic inputs in dendrites relative to the soma. Thus, spatially clustered glutamatergic synaptic inputs arriving synchronously can depolarize dendrites sufficiently to unblock the voltage-sensitive NMDA channels to fire “NMDA spikes” (Schiller et al., 2000).

The authors consider how the propagation of NMDA spike across the neuron affects plasticity. Synaptic NMDA receptors conduct calcium only in the presence of glutamatergic input. However, as the signal propagates through the neuron, voltage-gated calcium channels (VGCCs) can also conduct calcium without requiring a ligand, and in proportion to the voltage at their position. They investigate plasticity by implementing a model synapse from the Blue Brain Project (Chindemi et al., 2022). This model, based on the calcium-control hypothesis, posits either no plasticity, depression, or potentiation with increasing concentration of synaptic calcium. Thus, synapses receiving direct input (homosynaptic) may get modified based on the calcium influx via NMDA receptors, but other synapses on the dendritic tree (heterosynaptic) that “see” the propagating NMDA spike can also change because of calcium influx via VGCCs. Because of AVA, the NMDA spike decays more steeply toward the proximal than the distal direction. Thus, the degree and direction of heterosynaptic plasticity depends on the stimulus intensity and the dynamics of voltage propagation across the dendritic tree.

What is the significance of synaptic position in the context of heterosynaptic plasticity? First, Moldwin et al. (2023) discover that AVA leads to an asymmetry in calcium influx via VGCCs, which makes it much easier for the proximal inputs to heterosynaptically modify distal synapses than vice versa. They suggest that proximal synapses can thus act as “teaching signals'” for distal synapses.

Second, they analyze heterosynaptic plasticity with clustered and distributed inputs. Higher input resistance toward distal dendrites has the consequence that relatively smaller inputs can produce a large depolarization i.e., the effective threshold for dendritic spikes is lower distally than perisomatically. AVA works in the opposite direction to make inputs easier to propagate from soma to distal positions. As the net outcome, heterosynaptic plasticity can be induced more efficaciously if inactive synapses are 'sandwiched’ between active distal and proximal clusters. This suggests a coincidence threshold or an “AND”-type computation for distal and proximal inputs for heterosynaptic plasticity.

Third, two distal branches can compete such that if one homosynaptically potentiates, the other is heterosynaptically depressed. As input on one distal branch propagates down to the branching point, and back up on the competing distal branch, the voltage decays in the sister branch such that the VGCCs can only bring in enough calcium to induce depression, but not potentiation. This makes distal dendrites an attractive substrate for mutual-inhibition or winner-takes-all computation for plasticity. Put together, synaptic input clusters can be thought of as having heterosynaptic influence-maps on the neuron based on stimulus intensity, spatial position, and the specific parameters of the plasticity rule.

This study raises several interesting questions. How do heterosynaptic plasticity influence-maps change for different neuronal morphologies? Pyramidal neurons are generally characterized by short basal dendrites and a distal tuft connected to an apical dendrite. But there are pronounced differences among neuron types in the brain, and even between pyramidal neurons from different regions of the cortex and the hippocampus (Spruston, 2008). Second, the interaction between excitatory and inhibitory synapses can change the effective depolarization and consequently, the calcium influx through VGCCs. There is clear domain-specific targeting of inhibition from different interneuron types in pyramidal neurons, e.g., parvalbumin-type interneurons tend to target perisomatically and somatostatin-type interneurons target distal dendrites (Bloss et al., 2016). How does heterosynaptic plasticity affect this spatial organization of inputs? Similarly, calcium concentration can be affected by intracellular stores and neuromodulators, and intrinsic plasticity can modify non-synaptic ion channels. Branches could thus be made differentially permissive for voltage or their gain for calcium influx could be individually tuned. Finally, what do these mechanisms mean for behavior and learning?

Investigating these remarkable sub-neuronal interactions provide insights about the diverse computational possibilities available to neurons. Some spiking neural network frameworks such as “Dendrify” have begun to incorporate these insights (Pagkalos et al., 2023). These steps pave the way forward to a deeper understanding of the brain and potentially better machine learning.

## Synthesis

Reviewing Editor: Arvind Kumar, KTH Royal Institute of Technology
